# Quality assurance in MRI breast screening: comparing signal-to-noise ratio in dynamic contrast-enhanced imaging protocols

**DOI:** 10.1088/0031-9155/61/1/37

**Published:** 2015-11-25

**Authors:** Evanthia Kousi, Marco Borri, Jamie Dean, Rafal Panek, Erica Scurr, Martin O Leach, Maria A Schmidt

**Affiliations:** CR-UK and EPSRC Imaging Centre, Royal Marsden NHS Foundation Trust and Institute of Cancer Research, Sutton, Surrey, SM2 5PT, UK; Eva.Kousi@icr.ac.uk; Marco.Borri@icr.ac.uk; Jamie.Dean@icr.ac.uk; Rafal.Panek@icr.ac.uk; Erica.Scurr@rmh.nhs.uk; Martin.Leach@icr.ac.uk; Maria.Schmidt@icr.ac.uk

**Keywords:** MRI, quality assurance, breast, DCE-MRI, SNR

## Abstract

MRI has been extensively used in breast cancer staging, management and high risk screening. Detection sensitivity is paramount in breast screening, but variations of signal-to-noise ratio (SNR) as a function of position are often overlooked. We propose and demonstrate practical methods to assess spatial SNR variations in dynamic contrast-enhanced (DCE) breast examinations and apply those methods to different protocols and systems.

Four different protocols in three different MRI systems (1.5 and 3.0 T) with receiver coils of different design were employed on oil-filled test objects with and without uniformity filters. Twenty 3D datasets were acquired with each protocol; each dataset was acquired in under 60 s, thus complying with current breast DCE guidelines. In addition to the standard SNR calculated on a pixel-by-pixel basis, we propose other regional indices considering the mean and standard deviation of the signal over a small sub-region centred on each pixel. These regional indices include effects of the spatial variation of coil sensitivity and other structured artefacts.

The proposed regional SNR indices demonstrate spatial variations in SNR as well as the presence of artefacts and sensitivity variations, which are otherwise difficult to quantify and might be overlooked in a clinical setting. Spatial variations in SNR depend on protocol choice and hardware characteristics. The use of uniformity filters was shown to lead to a rise of SNR values, altering the noise distribution. Correlation between noise in adjacent pixels was associated with data truncation along the phase encoding direction.

Methods to characterise spatial SNR variations using regional information were demonstrated, with implications for quality assurance in breast screening and multi-centre trials.

## Introduction

Magnetic resonance imaging (MRI) has been extensively used in breast cancer staging, management and high risk screening (DeMartini *et al* 2008, Morrow *et al* 2011). Current American and European radiology guidelines recommended MRI for screening women at high risk of developing breast cancer due to its high sensitivity compared to x-ray mammography (National Institute for Health and Clinical Excellence 2006, Saslow *et al* 2007, Calonge *et al* 2009, Lee *et al* 2010). Breast MRI protocols include anatomical *T*_1_ and *T*_2_ weighted imaging and dynamic contrast enhanced (DCE) MRI (American College of Radiology 2003, Clayton 2012) with analysis and classification of the time-signal intensity curve to characterise the pattern of uptake and washout of paramagnetic contrast agents (Eyal and Degani 2009). There is a clear need for quality assurance (QA) in screening programmes for surveillance of women at high risk of developing breast cancer. In the UK the National Health Service Breast Screening Programme (NHSBSP) recommend weekly testing of signal-to-noise ratio (SNR) and water/fat suppression effectiveness, using techniques that are sensitive to individual coil element failures (Clayton *et al* 2012).

Traditional SNR measurements do not provide information on spatial variations of SNR (Price *et al* 1990, Gudbjartsson and Patz 1995, Lerski *et al* 1998, Ihalainen *et al* 2011), and presume the noise distribution is approximately Gaussian or Ricean (Gudbjartsson and Patz 1995) to calculate a figure that relates to a region of interest. Current MRI techniques, in contrast, make use of phased-array coils, parallel imaging techniques and uniformity filters (Pruessmann *et al* 1999, Griswold *et al* 2002, Lin *et al* 2004). Correlation between the noise measured with different phased array elements has been demonstrated (Constantinides *et al* 1997) and, in addition, parallel imaging techniques were shown to affect noise distribution (Dietrich *et al* 2007). SNR is thus expected to be position dependent, and dependent on pulse sequence parameters. It is therefore desirable to characterise SNR over the entire volume occupied by the breasts within the coil, for the pulse sequences employed in DCE examinations.

Mapping SNR as a function of position is most common in neurology applications, as the fast imaging sequences enable the acquisition of a large number of images within a short period. Several authors propose processing the ratio of mean and standard deviation of image intensity for each voxel to provide SNR maps (Price *et al* 1990, Chen *et al* 2004, Friedman and Glover 2006). SNR is thus characterised using specific sequences employed clinically, and the results have direct relevance to those applications. This approach is most informative if a large enough number of images is used to characterise the noise distribution. However, high resolution 3D datasets are acquired in approximately 1 min for breast DCE (Clayton *et al* 2012), and it is not practical to acquire more than 20–30 datasets for QA purposes. Other alternative approaches require access to raw data and reconstruction algorithms, and therefore their use is not widespread (Robson *et al* 2008).

There is considerable discussion within the literature on the merits of different approaches to developing breast DCE protocols to comply with breast screening guidelines while ensuring correct assessment of contrast agent uptake curves (Fan *et al* 2007, Schabel and Parker 2008, Jansen *et al* 2009, De Naeyer *et al* 2011, Freed 2012, Ledger *et al* 2014); in this discussion, the SNR dependency on position is often overlooked. This article proposes practical methods to characterise the spatial variation of SNR in breast DCE protocols, considering the hardware (breast coil and receiver chain) in conjunction with the chosen pulse sequence. These methods were applied to different systems and protocols used within our institution, with breast coils of different designs, and employed to investigate patterns of SNR variation as a function of position.

## Methods

### Data acquisition

Over the past three years, our institution has performed breast MRI examinations both at 1.5 T (Siemens MAGNETOM Avanto, Siemens MAGNETOM Aera) and 3.0 T (Siemens MAGNETOM Skyra), employing dedicated breast coils of different design (4 to 18 element arrays). In this article we employ data from these systems to propose and demonstrate QA methods for general use; the hardware is not described in detail because methodological developments are our main concern. All DCE protocols employ 3D, fat-suppressed, spoiled gradient-echo pulse sequences, with readout gradient in the anterior/posterior direction in a transaxial volume. Fat-suppression is attained using the SPAIR (SPectral Attenuated Inversion-Recovery) technique. All protocols use parallel imaging techniques with at least an acceleration factor of two (left/right direction). All protocols employ truncation of the data matrix, and/or partial Fourier acquisitions to acquire a complete high-resolution 3D dataset within 1 min, thus complying with current breast DCE guidelines (Clayton *et al* 2012). Basic protocol characteristics are shown in table 1. No view sharing techniques were employed in any protocol.

**Table 1.** Acquisition parameters of the different breast protocols.

Scanner	System I	System II	System III	System III
	Protocol I	Protocol II	Protocol IIIa	Protocol IIIb
Field strength (T)	1.5	1.5	3	3
TR (ms)	4.5	4.99	5.07	5.07
TE (ms)	2	2.25	1.68	1.69
Acquisition Time (s)	56	60	58	53
Flip angle (°)	18	18	18	18
Number of slices	160	160	160	160
Pixel size (mm^2^)	1.31 × 1.31	1.31 × 1.31	0.88 × 0.88	0.94 × 0.94
Slice thickness (mm)	1	1	1	1
Reconstruction matrix (A/P × L/R)	320 × 290	320 × 290	384 × 326	384 × 326
Acquistion matrix (A/P × L/R)	320 × 218	320 × 218	384 × 280	384 × 329
Bandwidth (Hz/px)	390	319	385	385
Phase and slice partial Fourier	6/8 and 6/8	6/8 and 6/8	7/8 and 6/8	7/8 and 6/8
Parallel imaging factor	2	2	3	3
Parallel imaging direction	L/R GRAPPA	L/R GRAPPA	L/R GRAPPA	L/R CAIPI
Coil description	Biopsy-Compatible coil with adjustable coil geometry	1st generation dedicated breast coil	Breast coil with rigid coil geometry	Breast coil with rigid coil geometry
Number of coil elements	8	4	18	18

In order to compare breast DCE examinations, all protocols were employed to scan two uniform oil-filled cylindrical test objects. Quantitative SNR measurements were undertaken on iso-paraffinic oil (Bayol 35 Oil, 11 cm diameter test object) of *T*_1_ 165 ms and 220 ms at 1.5 T and 3.0 T, respectively. Those *T*_1_ values are comparable to an enhancing lesion, following an injection of a single dose of contrast agent (Cron *et al* 2004). Bottles were strapped to the coil and patient couch to minimise mechanical vibration and left in place for at least 30 min prior to measurements. For each DCE examination 20 separate 3D datasets were acquired within 20 min. Fat suppression was disabled by setting the SPAIR RF power to zero for all test object scans. Disabling fat suppression did not affect the timing of the DCE sequences used. Images were acquired with and without the uniformity filter provided by the manufacturer, which is employed in all clinical examinations to reduce image intensity variations associated with coil sensitivity. The resulting set of images was processed off-line using in-house software (IDL 8.2, Boulder, USA).

In addition, a single volunteer was scanned in each system and a single dataset was acquired using the DCE sequence, without administration of contrast agent, with written consent and approval from the Local Ethics Committee. Automated shimming and measurement preparation was employed in all systems for this evaluation to reduce any potential operator dependency when images were compared.

### Data analysis

In this study each combination of coil and DCE protocol is characterised by a 4D dataset *A*(*i*, *j*, *k*, *t*), where *t* corresponds to the different time points ranging from 0 to *T*-1, and *A* is the image intensity for each position (*i*, *j*, *k*) in a 3D dataset. *T* corresponds to the total number of the datasets obtained. A calculation of the ratio of the mean signal to the mean standard deviation for each pixel produces a basic 3D map of SNR as a function of position (Price *et al* 1990, Lerski *et al* 1998), here referred to as SNR_0_:

1*a* \begin{eqnarray*}\text{SN}{{\text{R}}_{0}}(x,y,z)=\frac{\bar{A}(x,y,z)}{{{\sigma}_{A}}(x,y,z)},\end{eqnarray*}

where

1*b* \begin{eqnarray*}\bar{A}(x,y,z)=\frac{1}{T}\underset{t=0}{\overset{T-1}{\mathop \sum}}\,A\left(x,y,z,~t\right)\end{eqnarray*}

and

1*c* \begin{eqnarray*}{{\sigma}_{A}}(x,y,z)=\sqrt[2]{\frac{1}{(T-1)}\underset{t=0}{\overset{T-1}{\mathop \sum}}\,{{(A(x,y,z,~t)-\bar{A}(x,y,z))}^{2}}~~}.\end{eqnarray*}

We have limited the QA procedure to a 20 min data acquisition (i.e. *T*  =  20). In this article we propose practical methods to produce position dependent SNR maps using regional information, considering that 20 time points may not fully characterise the noise distribution for each voxel. For each voxel at given co-ordinates (*x*, *y*, *z*) we propose to make use of the statistical properties of the data contained within a 3D sub-region of dimensions *X*, *Y*, *Z*, in all *T* datasets.

For this purpose we define the set of images *B*(*x*, *y*, *z*, *t*) by subtracting the mean image intensity at each location:

2 \begin{eqnarray*}B(x,y,z,t)=A(x,y,z,t)-\bar{A}(x,y,z)\end{eqnarray*}

and define two additional SNR maps, SNR_R_ and SNR_A_:

3 \begin{eqnarray*}\text{SN}{{\text{R}}_{\text{R}}}(x,y,z)=\frac{\overline{{{A}_{r}}}(x,y,z)}{{{\sigma}_{\text{Br}}}(x,y,z)}\end{eqnarray*}

and

4 \begin{eqnarray*}\text{SN}{{\text{R}}_{\text{A}}}(x,y,z)=\frac{{{{\bar{A}}}_{r}}(x,y,z)}{{{\sigma}_{\text{Ar}}}(x,y,z)},\end{eqnarray*}

where

5*a* \begin{eqnarray*}{{\bar{A}}_{r}}(x,y,z)=\frac{1}{XYZT}\underset{t=0}{\overset{T-1}{\mathop \sum}}\,\underset{i=x}{\overset{x+X-1}{\mathop \sum}}\,\underset{j=y}{\overset{y+Y-1}{\mathop \sum}}\,\underset{k=z}{\overset{z+Z-1}{\mathop \sum}}\,A\left(i,j,k,~t\right),\end{eqnarray*}

5*b* \begin{eqnarray*}{{\sigma}_{\text{Ar}}}(x,y,z)=\sqrt[2]{\frac{1}{(XYZT-1)}\underset{t=0}{\overset{T-1}{\mathop \sum}}\,\underset{i=x}{\overset{x+X-1}{\mathop \sum}}\,\underset{j=y}{\overset{y+Y-1}{\mathop \sum}}\,\underset{k=z}{\overset{z+Z-1}{\mathop \sum}}\,{{(A(i,j,k,~t)-{{{\bar{A}}}_{r}}(x,y,z))}^{2}}~~},\end{eqnarray*}

5*c* \begin{eqnarray*}{{\sigma}_{\text{Br}}}(x,y,z)=\sqrt[2]{\frac{1}{(XYZT-1)}\underset{t=0}{\overset{T-1}{\mathop \sum}}\,\underset{i=x}{\overset{x+X-1}{\mathop \sum}}\,\underset{j=y}{\overset{y+Y-1}{\mathop \sum}}\,\underset{k=z}{\overset{z+Z-1}{\mathop \sum}}\,{{(B(i,j,k,~t)-{{{\bar{B}}}_{r}}(x,y,z))}^{2}}~~},\end{eqnarray*}

and

5*d*  \begin{eqnarray*}{{\bar{B}}_{r}}(x,y,z)=\frac{1}{XYZT}\underset{t=0}{\overset{T-1}{\mathop \sum}}\,\underset{i=x}{\overset{x+X-1}{\mathop \sum}}\,\underset{j=y}{\overset{y+Y-1}{\mathop \sum}}\,\underset{k=z}{\overset{z+Z-1}{\mathop \sum}}\,B\left(i,j,k,~t\right)=0.\end{eqnarray*}

${{\bar{A}}_{r}}$ and ${{\bar{B}}_{r}}$ are the mean values of the functions *A* and *B* within the local sub-region, and ${{\sigma}_{\text{Ar}}}$, ${{\sigma}_{\text{Br}}}$ are the standard deviation values for the non-subtracted (*A*) and the subtracted (B) datasets, respectively. We propose that SNR_R_ will provide a better description of the variation of SNR as a function of position than SNR_0_, for relatively small 3D sub-regions (low values of *X*, *Y*, *Z*), as the local noise distribution will be characterised by a larger number of pixels. In effect, a box function of dimensions *X*, *Y* and *Z* is employed as a kernel—thus allowing a trade-off between higher spatial resolution in SNR maps and adequate noise characterisation. We employ sub-regions containing at least 6 voxels (2  ×  3  ×  1) to ensure that at least 120 points are used to characterise the noise distribution at each location (for *T*  =  20). In contrast with SNR_R_, SNR_A_ includes—in the standard deviation ${{\sigma}_{\text{Ar}}}$—signal variations associated not only with noise but also with spatial variations of coil sensitivity and any other artefacts. We thus hypothesize that the index SNR_A_ will allow for a comprehensive assessment of both protocol and coil performance. This approach presumes there is no drift in image intensity over time throughout the acquisition of the *T*  =  20 images, and this hypothesis was checked for each dataset prior to further processing by measuring the image intensity over a central region of interest over time.

DCE data are acquired with parallel imaging, employing a combination of signals from different coil elements, and some data truncation. Therefore it would be incorrect to presume spatially uncorrelated noise within a given local region (Dietrich *et al* 2007). The spatial auto-correlation function was therefore calculated in order to determine the best approach to choosing regions of interest to calculate regional SNR indices (SNR_R_ and SNR_A_), i.e. the average Pearson correlation coefficient was calculated to assess the correlation between the time evolution of the image intensity of each voxel and its neighbours.

A standard SNR measurement to characterise the different coils and protocols was used as a reference; employing a small central region (comprising 300 voxels) placed in the position corresponding to the centre of the breast in the most central transaxial slice. SNR within the central region was also normalised to voxel size, total acquisition time and acquisition bandwidth to facilitate comparison between systems. For the purposes of normalisation, a bandwidth of 30 kHz was arbitrarily chosen as a reference (Dietrich *et al* 2007).

## Results

**Figure 1.**
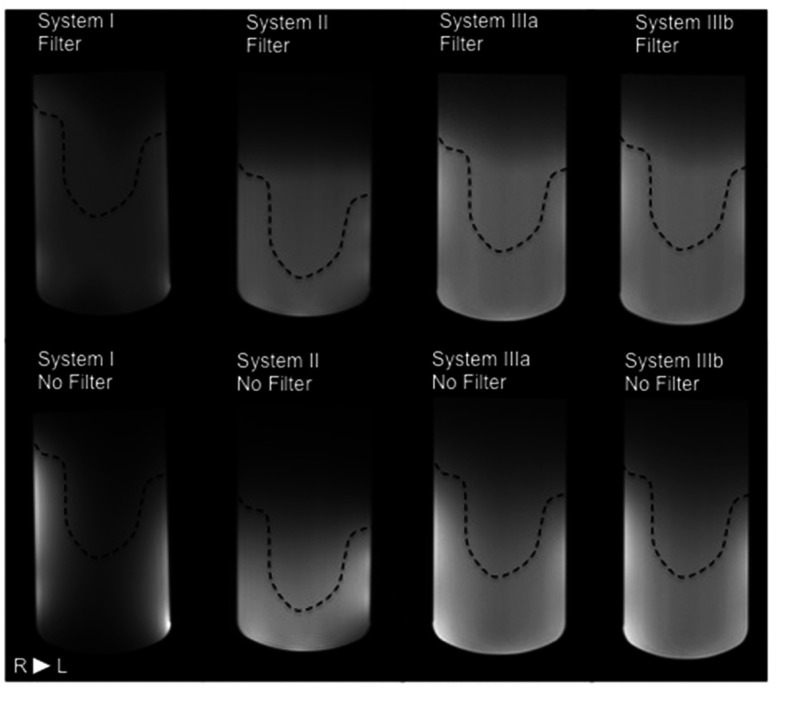
Transaxial slice (T_1_-W, 3D GRE) at the centre of the test object images acquired with (top row) and without (bottom row) uniformity filters for each system. Coil volume occupied by the breasts is different for different coil designs (dashed right-breast contours). The right side of the breast coil is shown for each system, with the axilla region to the left of the images (patient right). Good overall image quality is demonstrated. Different patterns of variation of signal intensity as a function of position are visible, particularly at the most anterior region (bottom of images). Window levels are kept the same for images with/without filters.

Figure 1 shows transaxial slices at the centre of the test object images acquired in each system, covering the right half of the breast coil. Images with and without uniformity filters (top and bottom row, respectively) demonstrate good overall image quality and different patterns of image intensity variation as a function of position.

**Figure 2.**
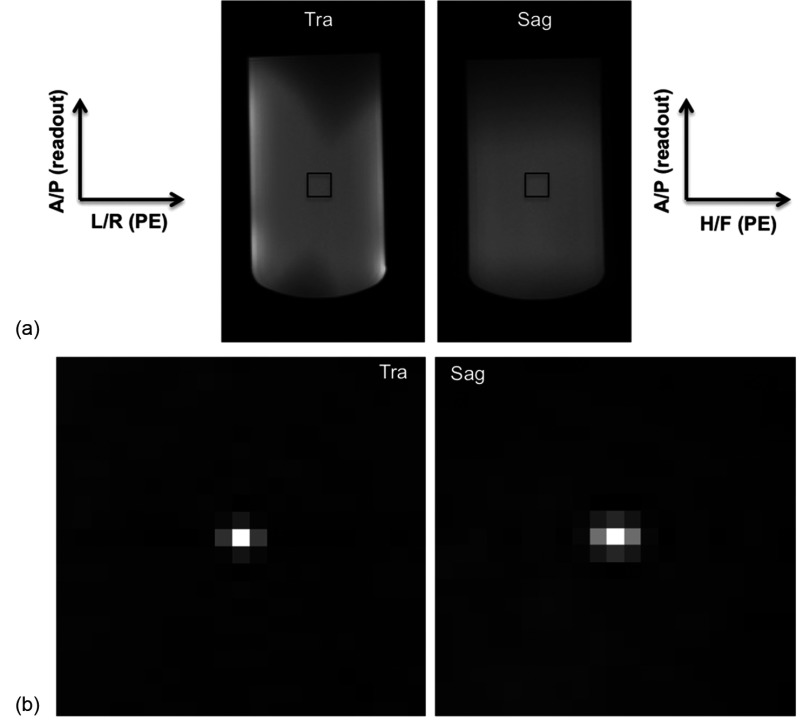
(a) Transaxial (left) and sagittal (right) images at the centre of the test object, acquired with system I. (b) Spatial autocorrelation maps for the central ROI indicated, in the transaxial and sagittal planes. Readout direction is vertical in all pictures. Higher correlations between noise in adjacent pixels along the PE directions is observed.

Figure 2(a) shows transversal and sagittal sections through the centre of the 3D volume corresponding to the right side of the breast coil for system I. Figure 2(b) shows the spatial autocorrelation for a 21  ×  21 pixel central region, demonstrating correlation between the noise in adjacent pixels, with higher correlation between adjacent pixels along the phase encoding directions (R/L and H/F), compared to the readout direction (A/P).

This pattern is reproduced for each of the system/protocol combination shown in table 1, as all employ readout gradient along A/P direction, irrespective of the use of uniformity filters. The image intensity was measured over the same 21  ×  21 central ROI for each data set and the variations were found to be smaller than 0.3% for all protocols.

**Figure 3.**
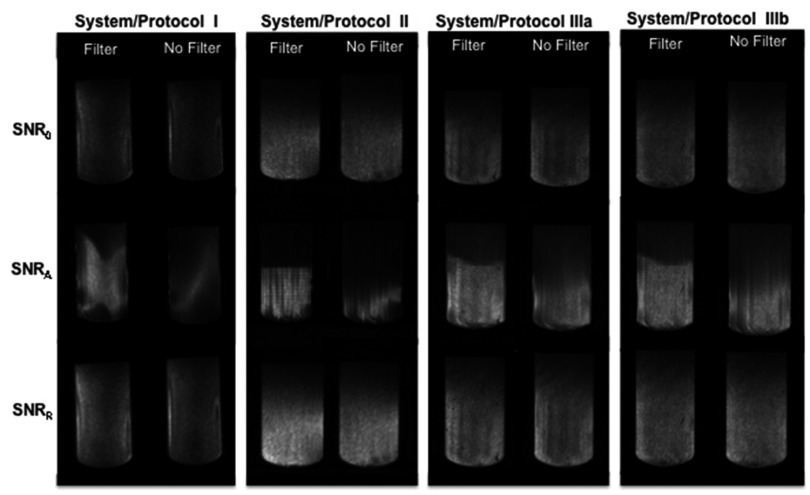
SNR_0_, SNR_A_ and SNR_R_ maps calculated for each system/protocol with (left) and without (right) the uniformity filters for the same test object (transaxial view at the centre of the coils). The window levels are kept the same for each system, showing that images acquired with the uniformity filter have higher SNR values. The calculation of SNR_A_ makes many artefacts more readily visible. The uniformity filter appears to have introduced a structured noise to images in the anterior–posterior direction acquired in systems I and II, visible in the SNR_A_ maps.

Figure 3 shows the maps SNR_0_, SNR_A_ and SNR_R_ for each system, calculated from images acquired with and without the uniformity filters used in the clinical protocols, employing a 3  ×  2  ×  1 pixels sub-region (in directions A/P, L/R, H/F, respectively) for SNR_A_ and SNR_R_ maps.

SNR_R_ maps show spatial variations that depend on the system and protocol employed; protocols IIIa and IIIb on the same system (and same receiver coil) produce different results. In addition SNR_A_ demonstrates the presence of many structured artefacts and coil dependent variations of signal intensity in each system. Regions close to coil elements are highlighted in all systems, and faint vertical bands become visible. The uniformity filters introduced structured artefacts in the anterior–posterior direction to images acquired in systems I and II.

Values of SNR for the central region are presented in table 2, for data acquired with and without uniformity filters.

**Table 2.** SNR measurements (mean  ±  standard deviation) within region of interest (300 pixels) at the centre of breast position in the transaxial plane using all three SNR calculation approaches (SNR_0_, SNR_A_, SNR_R_) for each protocol and breast coil design. Normalized SNR_R_ (NSNR_R_) values are also shown.

System	I	I	II	II	IIIa	IIIa	IIIb	IIIb
Uniformity filter	YES	NO	YES	NO	YES	NO	YES	NO
SNR_0_	190 ± 32	160 ± 27	490 ± 95	430 ± 64	250 ± 51	220 ± 33	240 ± 41	210 ± 41
SNR_A_	180 ± 19	140 ± 18	370 ± 80	120 ± 28	240 ± 28	140 ± 17	230 ± 2 6	130 ± 15
SNR_R_	190 ± 21	160 ± 16	490 ± 53	420 ± 38	250 ± 30	210 ± 22	240 ± 27	210 ± 23
NSNR_R_ (1000 cm^−3^ s^−1/2^)	30	25	68	59	92	79	80	72

The difference between values of SNR_0_ and SNR_R_ for the central region is under 5%, and SNR_A_ is lower than SNR_R_ and SNR_0_ as expected. The difference between SNR_R_ and SNR_A_ at the central region is particularly large for system II (25%). Values of SNR_R_ normalised for voxel size and receiver bandwidth are also shown for comparison.

**Figure 4.**
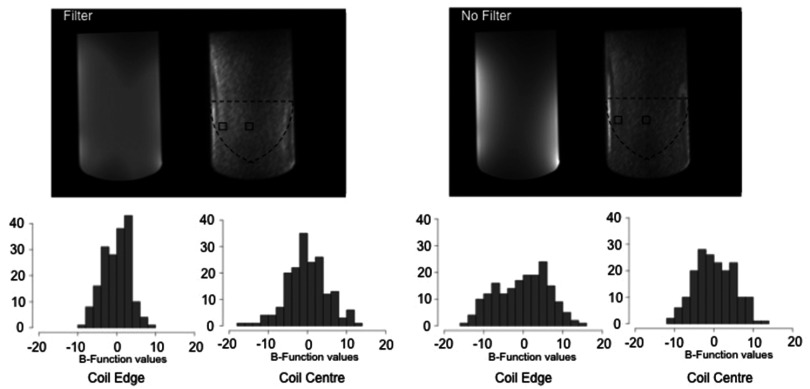
Transversal image at the centre of a uniform test object and SNR_R_ map, acquired with and without uniformity filters with protocol I (top). Function B ($B(x,y,z,t)=A(x,y,z,t)-\bar{A}(x,y,z)$ for the two regions of interest indicated, for transverse images acquired with (left) and without (right) uniformity filters in system I (bottom). Regions of interest consist of 3  ×  3  ×  20 pixels, and histograms are centred around zero, as expected. The introduction of the uniformity filters results in changes to the noise distribution as shown at two different locations (edge, centre). The narrower distributions on images acquired with the uniformity filter lead to a higher SNR.

Table 2 also indicates that the use of uniformity filters have a large impact on calculated SNR values; the introduction of filters increased SNR values by 15% on average in systems I, II and III. Figure 4 shows a histogram of values of the function B within small regions at different locations for two sets of images acquired with and without filters, using the same system and protocol, demonstrating a change to the distribution of values.

**Figure 5.**
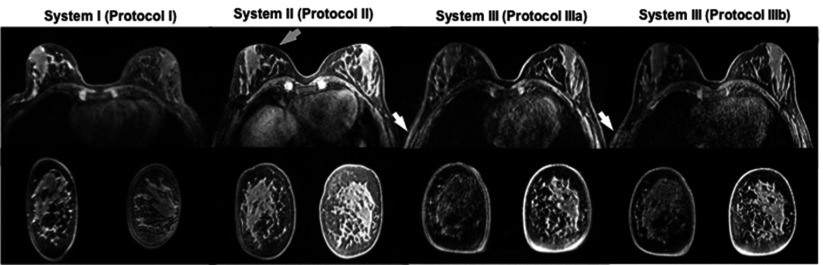
Breast images of the same volunteer acquired with different systems and protocols. Window levels were adjusted independently for each dataset to enable best visualisation of the breast parenchyma. The receiver coils used with each protocol place the breasts in different positions, with implications for shimming and image quality. Protocols I and II at 1.5 T are very similar, but produce very different clinical images. The structured artefact in the anterior–posterior direction demonstrated in figure 3 for those systems is barely visible (grey arrow) and would have been overlooked in a clinical setting. Protocols IIIa and IIIb were employed in the same 3 T system, but with different parallel imaging techniques. IIIb has superior spatial resolution, but images appear noisier in the axilla region (white arrows).

Figure 5 shows breast images of the same volunteer acquired with different protocols/systems. Fat suppression efficiency is variable with automated shimming over the whole imaging volume. Breasts are deformed differently to fit different coils, a confounding factor in image quality. Protocols I and II are very similar (1.5 T, same voxel size), but produce very different results—protocol II produced sharper images, showing smaller details. Protocols IIIa and IIIb were applied to the same 3.0 T system and breast coil, and again the images differ in quality. Protocol IIIb demonstrates smaller details within the breast paranchyma and there are fewer truncation artefacts, but images appear considerably noisier at the axilla.

## Discussion

In this article we proposed and demonstrated methods to characterise the SNR spatial dependency for breast DCE protocols with a simple and relatively fast procedure. Drifts in image intensity over time were not expected and were not found, therefore it is not necessary to post-process images to remove long-term variations in image intensity as proposed by Friedman (Friedman and Glover 2006). Three types of SNR maps were calculated for all systems: SNR_0,_ SNR_R_ and SNR_A_. SNR_0_ and SNR_R_ have similar characteristics as expected, but the ability to trade spatial resolution for a better noise characterisation is advantageous when working with small datasets. SNR_A_, in comparison, has provided additional information, highlighting a number of structured artefacts, not necessarily noticeable when one single dataset is scrutinised. Both data truncation and parallel imaging can give rise to structured artefacts (Dietrich *et al* 2007) which are not randomly distributed—their distribution is a function of the test object characteristics, and often replicates and distorts high contrast structures. These artefacts have an impact on image quality, but in a clinical setting the structured artefacts may be less conspicuous. For instance, the structured artefact associated with the use of uniformity filters, detected in the anterior–posterior direction by the calculation of SNR_A_ in system II, is barely visible (figure 5). Nevertheless, in DCE-MRI, signal and noise instabilities over the whole acquisition period—captured in SNR_A_—could significantly affect the shape of the enhancement curves and hence their diagnostic performance. Furthermore many artefacts detected with the calculation of SNR_A_ could be mistakenly attributed to subject motion, which is clearly undesirable. It is interesting to notice that the difference between values of SNR_R_ and SNR_A_ in the central region is largest on our oldest system (system II). Assessment of artefacts is an important part of the characterisation of breast protocols, and the calculation of SNR_A_ is a sensitive method.

This article demonstrates many confounding factors relating to SNR measurements: spatially correlated noise (figure 2) and position dependent noise distribution (figure 4), affected by the uniformity filters. Considering our results for all systems, it is advantageous to extend the sub-region in the readout direction to calculate SNR_R_ and SNR_A,_ as the correlation between noise in adjacent voxels is smaller.

Optimisation of DCE breast protocols involves finding a suitable compromise between spatial and temporal resolution while ensuring that the image intensity remains proportional to contrast agent concentration. In our institution different approaches were used and, although all protocols comply with the current guidelines, they have different resolution and achieve different image quality, as a result of differences in the field strength and coil geometry.

It is possible that the SNR measurements performed are sensitive to vibration, and great care was taken to minimize it. This is not necessarily a drawback for this type of testing, as patient breasts are not rigid. We have repeated SNR measurements with a gel test object in one of our systems, and obtained similar results.

The SNR measurements performed on a central region suggest that the 4-element coil of rigid geometry (system II) provides a better performance than the modern biopsy-compatible coil (system I). Although this is likely, it is also possible that system II simply performs better on the test object. In practice breast patients are of different shapes and sizes, and we have not addressed fully the impact of coil design on image quality considering the high variability in filling factors within the patient population. The receiver coils deform the breast in different ways, and this in turn has an impact on shimming—as clearly demonstrated by the imperfect automated fat suppression on volunteer images.

The normalised SNR values presented in table 2 must be treated with caution: we acknowledge we are comparing images acquired with different parallel imaging factors (and different spatial resolution), which may be differently affected by the confounding factors already discussed. The shortcomings of the normalisation process were discussed elsewhere and are pertinent to our work (Erdogmus *et al* 2004). Although it appears that the SNR gains at 3.0 T (protocol III) are modest when compared to 1.5 T (protocols I and II), in reality the 3.0 T protocols were set up for higher spatial resolution, and the normalisation process may not necessarily account fully for that. For a direct comparison of hardware performance, it may be more productive to use the same basic protocol in all systems, without parallel imaging and filters. The approach we propose goes beyond hardware performance and characterises the protocol as a whole, including hardware, pulse sequence design and the prevalent use of uniformity filters. These measurements can be used for system performance assessment on a long term basis. In particular, the proposed index SNR_A_ has drawn attention to artefacts that could have remained undetected, and is therefore a useful tool in QA. The methods we propose are therefore invaluable in longitudinal studies and clinical trials; they can be used to detect deterioration of a system performance over time or to stipulate a minimum SNR value over a given volume for trials which employ different hardware, or even different DCE protocols in different systems. For instance, regions with low SNR values could be identified and carefully monitored over time. Quality assurance should require stable values of SNR_A_ and a minimum value of SNR over the coil volume to be occupied by the breasts.

In conclusion, we proposed and demonstrated a method to acquire and process data to map SNR in breast DCE protocols, making use of regional information to characterise each voxel. This work has demonstrated artefacts and highlighted a number of confounding factors in the SNR measurements, with implications for quality assurance in multi-centre trials and breast cancer screening services, which benefit from standardisation.
